# SBRT for centrally localized NSCLC – What is too central?

**DOI:** 10.1186/s13014-016-0732-5

**Published:** 2016-12-03

**Authors:** J. Roesch, C. Panje, F. Sterzing, F. Mantel, U. Nestle, N. Andratschke, M. Guckenberger

**Affiliations:** 1 Department of Radiation Oncology, Universitässpital Zürich, Zürich, Switzerland; 2 Heidelberg Institute of Radiation Oncology (HIRO), Heidelberg, Germany; 3 Department of Radiation Oncology, Universitätsklinikum Würzburg, Würzburg, Germany; 4 Department of Radiation Oncology, Universitätsklinikum Freiburg, Freiburg, Germany

**Keywords:** SBRT, SABR, NSCLC, Central lung, Pulmonary toxicity

## Abstract

**Purpose:**

Current guidelines recommend stereotactic body radiotherapy (SBRT) for stage I non-small-cell lung cancer (NSCLC) in medically inoperable patients. There are excellent outcome and toxicity data for SBRT of peripheral lung tumors. However, the discussion on SBRT for centrally located tumors is controversial. This study evaluated current clinical practice regarding SBRT of centrally located lung tumors, to identify common fractionation schedules and commonly accepted contraindications for SBRT.

**Methods:**

A questionnaire consisting of two parts was introduced at the annual meeting of the DEGRO working group on stereotactic radiotherapy, representing centers in Germany and Switzerland. The first part of the questionnaire covered general information about the centers, whereas the second part specifically addressed SBRT of centrally located lung tumors, using case examples of nine primary NSCLC patients. Reconstructions of a contrast enhanced CT, as well as PET-Imaging for each case were demonstrated to the participants.

**Results:**

Twenty-six centers participated in the meeting. The majority was academic (73%), participated in interdisciplinary thoracic oncology tumorboards (88%) and offered SBRT for lung tumors (96%). Two centers questioned the indication of SBRT for central lung tumors because of lack of evidence. The majority of centers had experience in SBRT for central lung tumors (88%) and half of the centers reported more than ten cases treated during a median period of five years. Most fractionation schedules used PTV encompassing doses of 48–60 Gy in eight fractions with maximum doses of 125–150%.

A clear indication for SBRT treatment was seen by more than 85% of centers in three of the nine patients in whom tumors were small and not closer than 2 cm to the main bronchus. Prior pneumonectomy or immediate adjacency to hilar/mediastinal structures were not considered as contraindications for SBRT. In cases where the tumor exceeded 4 cm in diameter or was located closer than 4 cm to the carina 50–80% of centers saw an indication for SBRT. One case, with a 7 cm tumor reaching to the carina would have been treated with SBRT only by one center.

**Conclusion:**

Within DEGRO working group on stereotactic radiotherapy, SBRT for small (<4 cm) early stage NSCLC is a common indication, if the minimal distance to the main bronchi is at least 2 cm. The controversy on the treatment of larger and more central tumors will hopefully be solved by ongoing prospective clinical trials.

## Background

Malignant neoplasms of the lung are the most frequent cause of cancer-related death in the world with approximately 350,000 deaths in Europe and 1.6 million deaths worldwide in 2012 [1–3]. Although the overall prognosis of non-small cell lung cancer (NSCLC) is poor, early stage without regional or metastatic spread can be cured by local treatment. The combination of future screening programs on the basis of computed tomography (CT) and an aging population will most likely increase the incidence of early stage lung cancer, especially in elderly patients [4, 5].

Surgical lobectomy plus mediastinal lymph node dissection is the standard treatment for early stage NSCLC. However, the increasing number of elderly patients with comorbidities demonstrates the need for less-invasive therapies [6]. Stereotactic body radiotherapy (SBRT) for peripherally located early stage lung cancer has recently emerged as a safe and non-invasive alternative to surgical resection with equivalent rates of local tumor control, and has been established as standard of care for inoperable tumors in specialized centers [7–9].

However, centrally located lung tumors represent a challenge for both surgical treatment and SBRT. Central tumors may more likely require extensive surgery like pneumonectomy, which bears the risk of inferior outcome or inoperability [10, 11]. Likewise, there is evidence that SBRT of central tumors has an increased risk of severe toxicity, which may be up to 11 times higher than in peripheral tumors [12]. Overall, prospective data on SBRT of central tumors is sparse, but presently recruiting clinical trials will prospectively assess long term benefits and risks of SBRT for this patient population in order to generate solid evidence on local control and toxicity [13, 14].

Unfortunately, “central location” is not consistently defined in recent prospective trials, as shown in the following examples: (1) RTOG 0236: a tumor within 2 cm to the proximal bronchial tree (PBT), which was described as the distal 2 cm of the trachea, carina, main bronchi and named major lobar bronchi up to their first bifurcation (i.e. upper and lower lobe bronchi, intermedius bronchus, lingular bronchus) [15, 16]; (2) IASLC recommendation: a tumor within 2 cm to any mediastinal critical structure, including the bronchial tree, esophagus, heart, major vessels, spinal cord, brachial plexus, phrenic and recurrent laryngeal nerve [17]; (3) RTOG 0813: a tumor within 2 cm to the PBT or touching the mediastinal pleura [18]; some authors further enlarged the zone by tumors within 6 mm to the pericardium or 1 cm around the mediastinum [19, 20].

Furthermore, SBRT in daily routine follows not only published evidence but also depends on the physician’s experience and expertise, resulting in a variety of treatment schedules. At present, it is unknown whether SBRT can be applied to all centrally located tumors or whether there are locations which are too close to critical organs at risk.

Therefore, this study evaluated the patterns of current clinical practice of SBRT for centrally located lung tumors in 26 German and Swiss centers with the aim to identify common fractionation schedules and commonly accepted contraindications.

## Methods

A questionnaire consisting of two parts was presented to representatives of 26 centers in Germany (*n* = 25) and Switzerland (*n* = 1) during the annual meeting of the DEGRO working group on stereotactic radiotherapy. The first part of the questionnaire addressed general information about the centers such as type (academic vs non-academic), institution size, experience and treatment specifications regarding lung SBRT in general.

In the second part of the questionnaire, the participants were shown nine cases of centrally located primary NSCLC (cT2-4 cN0 cM0) and were asked to determine if SBRT was indicated and which fractionation schedule they would suggest. Furthermore, they were asked to provide reasons if they rejected SBRT in a specific case and to suggest alternative treatments. All presented cases originated from four different academic centers (UniversitätsSpital Zürich, Universitätsklinikum Würzburg, Universitätsklinikum Heidelberg, Klinikum der Universität München) and were consequently anonymized. The survey participants were shown axial, sagittal and coronal reconstructions of a contrast-enhanced 3D-CT of the lungs in combination with fluordeoxyglucose positron emission tomography (FDG-PET) imaging if available. The medical history for all cases was assumed to be identical: a 75-year-old male, active smoker with 25 pack-years and relevant comorbidities of chronic obstructive pulmonary disease (FEV_1_ = 1 l/s), diabetes type 2, hypertension and congestive heart disease after coronary dilatation because of unstable angina pectoris five years ago.

## Results

The majority of the participating centers were academic (*n* = 19), participated in interdisciplinary thoracic oncology tumorboards (*n* = 23) and practiced SBRT for lung tumors as standard (*n* = 25, see Table 1). Two centers questioned the suitability of SBRT for central lung tumors because of lack of evidence and therefore did not practice it. The majority of centers had experience in SBRT for central lung tumors. Thirteen centers referred more than ten treated cases during a median period of five years, whereas the remaining ten centers reported less than ten treated cases within a median period of three years.

**Table 1:** Individual center characteristics

General questionnaire items	
Center type	Academic = 19 Public = 4 Private = 2 Non specified = 1
Median number of linear accelerators	3 (range 2 – 7)
Regular participation in thoracic oncology tumorboard	88%
Overall experience in SBRT of the centrally located NSCLC	0 cases = 3 <10 cases = 10 >10 cases = 13
Duration of SBRT experience	5 years (range: 1 – 15)
General treatment items	
Imaging for target volume definition	4D-CT = 18 PET-CT = 21
Image guidance	CBCT = 24 TOMO = 2 Other = 2
Patient immobilization system	Body Fix/Frame = 11 vacuum matrass = 7 unknown = 7
Abdominal compression	3

A FDG-PET scan was an essential component of the staging procedure in each center and, when reasonable, was frequently used for target volume definition (*n* = 21, 81%). A 4D-CT scan was standard in 69% for treatment planning (*n* = 18). Image guidance by cone-beam CT or by another system was practiced by all centers. Most centers used eight fractions of 6–7.5 Gy prescribed to the 65-90% isodose surrounding the planning target volume (PTV) as their standard fractionation schedule for SBRT of peripherally and central lung tumors. About one quarter of the centers recommended three fractions of 12.5 Gy prescribed to an isodose line between 60-67%. No center used single fraction regimens as standard. Center characteristics and treatment standards are summarized in Tables 1 and 2, respectively.

**Table 2:** Most common standard fractionation schedules for SBRT of lung tumors in general

Fractions	Dose	Isodose line surrounding PTV	Number of centers	Percentage of centers
3	12.5	60–67%	6	26%
5	7	65–80%	2	9%
5	8	65%	1	4%
6	8	-	1	4%
8	6	65–80%	3	13%
8	7.5	70–90%	9	39%
10	6	90%	1	4%

**Table 3:** Cases grouped according to their level of acceptance

Level of acceptance	High (>85%)	Intermediate	Low (<50%)
Case Nr.	5, 6, 9	1, 2, 3, 7, 8	4
Most critical organ at risk	Main bronchus, esophagus, aorta	Main bronchus, upper lobe bronchus, aorta	Main bronchus
Maximal tumor diameter (range/median)	2.1–3.7 cm/3.5 cm	1.9–5.7 cm/4.7 cm	7.6 cm
Minimal distance to carina (range/median)	7.5–8.3 cm/7.8 cm	2.5–6.5 cm/3.8 cm	0 cm
Minimal distance to main bronchus (range/median)	1.9–4.6 cm/2.2 cm	0–0.5 cm/0 cm	0 cm
Alternative treatment	RT 70 Gy/35 fr.	RCT: 66 Gy/33 fr.	RT: 30 Gy/10 fr. or RCT: 66 Gy/33 fr.
Most frequently stereotactic fractionation	60 Gy/8 fr.	60 Gy/8 fr.	40 Gy/8 fr.
Second most frequently stereotactic fractionation	37,5–50 Gy/3 fr.	35–40 Gy/5 fr. 48 Gy/8 fr.	-

The following figures describe the tumor location and treatment recommendations for the nine cases. In the coronal (left-right), sagittal (anterior-posterior) and longitudinal (superior–inferior) axis the longest tumor diameters were measured. Furthermore, the maximum tumor diameter as well as the minimal distance to the carina, the mediastinum and the main bronchi are provided.

### Case 1

**Table:** 

Tumor location: Within left upper lobe, between left pulmonary artery and left upper lobe bronchus				
Shortest distance to mediastinum [cm]	Shortest distance to carina [cm]	Shortest distance to main bronchus [cm]	Size (cor/sag/long) [cm]	Maximum diameter [cm]
0	3.8	0	1.7/1.6/1.8	1.9
SBRT accepted	Most frequently used fractionation	Second most frequently used fractionation	Mean number of fractions	Mean dose per fraction [Gy]
21 (81%)	8 × 7.5 Gy @ 65–95%	8 × 6 Gy @ 65%	7.1	7.6
SBRT declined	Reasons to decline SBRT		Most frequently recommended alternative treatment	
5 (19%)	Risk to central airways		Conventionally fractionated RT to 70Gy	

Tumor characteristics of case 1. Abbreviations: *cor* coronal, *sag* sagittal, *long* longitudinal, *SBRT* Stereotactic body radiotherapy, *RT* Radiotherapy, *RCT* Radiochemotherapy

### Case 2

**Table:** 

Tumor location: Within left lower lobe, adjacent to main bronchus				
Shortest distance to mediastinum [cm]	Shortest distance to carina [cm]	Shortest distance to main bronchus [cm]	Size (cor/sag/long) [cm]	Maximum diameter [cm]
0	4.4	0	2.3/3.2/2.1	4.9
SBRT accepted	Most frequently used fractionation	Second most frequently used fractionation	Mean number of fractions	Mean dose per fraction [Gy]
20 (77%)	8 × 7.5 Gy @ 65–90%	5 × 7 Gy @ 65%	7.0	7.6
SBRT declined	Reasons to decline SBRT		Most frequently recommended alternative treatment	
6 (23%)	Size, risk for left main bronchus		Conventionally fractionated RT to 66-70Gy	

Tumor characteristics of case 2. Abbreviations: *cor* coronal, *sag* sagittal, *long* longitudinal, *SBRT* Stereotactic body radiotherapy, *RT* Radiotherapy, *RCT* Radiochemotherapy

### Case 3

**Table:** 

Tumor location: Centrally located within the right hilus				
Shortest distance to mediastinum [cm]	Shortest distance to carina [cm]	Shortest distance to main bronchus [cm]	Size (cor/sag/long) [cm]	Maximum diameter [cm]
0	2.5	0	3.2/3.5/3.2	3.5
SBRT accepted	Most frequently used fractionation	Second most frequently used fractionation	Mean number of fractions	Mean dose per fraction [Gy]
14 (54%)	8 × 7.5 Gy @ 80–90%	5 × 8 Gy @ 65%	7.5	6.3
SBRT declined	Reasons to decline SBRT		Most frequently recommended alternative treatment	
12 (46%)	Risk for right main bronchus		Conventionally fractionated RCT to 66 Gy	

Tumor characteristics of case 3. Abbreviations: *cor* coronal, *sag* sagittal, *long* longitudinal, *SBRT* Stereotactic body radiotherapy, *RT* Radiotherapy, *RCT* Radiochemotherapy

### Case 4

**Table:** 

Tumor location: large tumor reaching to carina, infiltrating the upper lobe bronchus and the main bronchus				
Shortest distance to mediastinum [cm]	Shortest distance to carina [cm]	Shortest distance to main bronchus [cm]	Size (cor/sag/long) [cm]	Maximum diameter [cm]
0	0	0	6.8/5.7/6.4	7.6
SBRT accepted	Most frequently used fractionation	Second most frequently used fractionation	Mean number of fractions	Mean dose per fraction [Gy]
1 (4%)	8 × 5 Gy	-	-	-
SBRT declined	Reasons to decline SBRT		Most frequently recommended alternative treatment	
25 (96%)	Size, risk to central airways		Conventionally fractionated RCT to 66Gy; Palliative RT 30 Gy/10 fr.	

Tumor characteristics of case 4. Abbreviations: *cor* coronal, *sag* sagittal, *long* longitudinal, *SBRT* Stereotactic body radiotherapy, *RT* Radiotherapy, *RCT* Radiochemotherapy

### Case 5

**Table:** 

Tumor location: centrally localized, next to right lower bronchus after pneumonectomy of the left lung				
Shortest distance to mediastinum [cm]	Shortest distance to carina [cm]	Shortest distance to main bronchus [cm]	Size (cor/sag/long) [cm]	Maximum diameter [cm]
3.5	7.8	1.9	3.5/2.8/2.5	3.5
SBRT accepted	Most frequently used fractionation	Second most frequently used fractionation	Mean number of fractions	Mean dose per fraction [Gy]
22 (88%)	8 × 7.5 Gy @ 65–90%	3 × 15 Gy @ 65%	6.0	9.4
SBRT declined	Reasons to decline SBRT		Most frequently recommended alternative treatment	
3 (12%)	Localization too central		Conventionally fractionated RT	

Tumor characteristics of case 5. Abbreviations: *cor* coronal, *sag* sagittal, *long* longitudinal, *SBRT* Stereotactic body radiotherapy, *RT* Radiotherapy, *RCT* Radiochemotherapy

### Case 6

**Table:** 

Tumor location: right lower lobe, adjacent to the esophagus				
Shortest distance to mediastinum [cm]	Shortest distance to carina [cm]	Shortest distance to main bronchus [cm]	Size (cor/sag/long) [cm]	Maximum diameter [cm]
0	7.5	2.2	2.5/2.3/1.8	3.7
SBRT accepted	Most frequently used fractionation	Second most frequently used fractionation	Mean number of fractions	Mean dose per fraction [Gy]
22 (88%)	8 x 7.5 Gy @ 65-90%	3 x 13.5/12,5 Gy @ 65%	6.8	8.5
SBRT declined	Reasons to decline SBRT		Most frequently recommended alternative treatment	
3 (12%)	Localization too central, risk to esophagus		Surgery, conventionally fractionated RT with small margins	

Tumor characteristics of case 6. Abbreviations: *cor* coronal, *sag* sagittal, *long* longitudinal, *SBRT* Stereotactic body radiotherapy, *RT* Radiotherapy, *RCT* Radiochemotherapy

### Case 7

**Table:** 

Tumor location: Left upper lobe, Adjacent to the mediastinum at the height of the aortic arch				
Shortest distance to mediastinum [cm]	Shortest distance to carina [cm]	Shortest distance to main bronchus [cm]	Size (cor/sag/long) [cm]	Maximum diameter [cm]
0	6.5	5	2.0/3.9/4.7	4.7
SBRT accepted	Most frequently used fractionation	Second most frequently used fractionation	Mean number of fractions	Mean dose per fraction [Gy]
21 (81%)	8 x 7.5 Gy @ 65-80%	8 x 6 Gy @ 65-80%	6.8	8.9
SBRT declined	Reasons to decline SBRT		Most frequently recommended alternative treatment	
5 (19%)	Risk for aorta		Conventionally fractionated RT +/− chemotherapy	

Tumor characteristics of case 7. Abbreviations: *cor* coronal, *sag* sagittal, *long* longitudinal, *SBRT* Stereotactic body radiotherapy, *RT* Radiotherapy, *RCT* Radiochemotherapy

### Case 8

**Table:** 

Tumor location: Large in size, right lower lobe, right after ramification of intermediate bronchi				
Shortest distance to mediastinum [cm]	Shortest distance to carina [cm]	Shortest distance to main bronchus [cm]	Size (cor/sag/long) [cm]	Maximum diameter [cm]
0	3.6	0.5	4.5/5.6/5.0	5.7
SBRT accepted	Most frequently used fractionation	Second most frequently used fractionation	Mean number of fractions	Mean dose per fraction [Gy]
16 (62%)	8 x 7.5 Gy @ 65-85%	5 x 7 Gy @ 60-65%	7.3	7.6
SBRT declined	Reasons to decline SBRT		Most frequently recommended alternative treatment	
10 (38%)	Size		Conventionally fractionated RT, Palliative hypofractionated RT	

Tumor characteristics of case 8. Abbreviations: *cor* coronal, *sag* sagittal, *long* longitudinal, *SBRT* Stereotactic body radiotherapy, *RT* Radiotherapy, *RCT* Radiochemotherapy

### Case 9

**Table:** 

Tumor location: left lower lobe, adjacent to Aorta				
Shortest distance to mediastinum [cm]	Shortest distance to carina [cm]	Shortest distance to main bronchus 2[cm]	Size (cor/sag/long) [cm]	Maximum diameter [cm]
0	8.3	4.6	1.8/1.2/1.5	2.1
SBRT accepted	Most frequently used fractionation	Second most frequently used fractionation	Mean number of fractions	Mean dose per fraction [Gy]
22 (85%)	8 x 7.5 Gy @ 65-90%	3 x 12,5 Gy @ 60-65%	6.6	8.7
SBRT declined	Reasons to decline SBRT		Most frequently recommended alternative treatment	
4 (15%)	Risk for aorta		Palliative RT, surgery	

Tumor characteristics of case 9. Abbreviations: *cor* coronal, *sag* sagittal, *long* longitudinal, *SBRT* Stereotactic body radiotherapy, *RT* Radiotherapy, *RCT* Radiochemotherapy

## Discussion

This survey demonstrates the current controversy regarding indications and contraindications for SBRT in central lung tumors, with a clear tendency towards the use of risk-adapted irradiation schedules. We categorized ﻿cases according to their level of acceptance (see Table 3) and identified three distinct decision criteria for SBRT: tumor size, exact location within the central zone and relation to the surrounding critical organs at risk, particularly the central airways.

We observed a high agreement (>85%) to perform SBRT despite central location for tumors with a maximal diameter of up to 4 cm, a minimal distance to the main bronchi of about 2 cm and without infiltration of lobar bronchi.

These tumors were located within the central zone as defined in the RTOG 0813 trial, but mainly distally with regard to the original definition by Timmerman et al. [12, 18]. The latter was used in the RTOG 0236 trial where the tumor was required to have a distance of at least 2 cm to the PBT, which was defined as the trachea, carina, main bronchi, and named major lobar bronchi up to their first bifurcation (i.e. upper and lower lobe bronchi, intermedius bronchus, lingular bronchus) [15, 16]. RTOG 0813 included tumors within 2 cm to the PBT or touching the mediastinum [18]. Furthermore, case 5 showed that prior pneumonectomy was not considered as a contraindication even in centrally located tumors, offering these high-risk patients a potentially curative treatment option.

Lung tumors which had only an intermediate level of acceptance for SBRT (54-81%) were located within the central zone as defined in RTOG 0813. SBRT was contraindicated by several centers due to close proximity to the central airways (case 1–3), particularly when the tumor was directly located at the hilus (case 2). These cases met the criteria of “ultra-central” tumors which can be defined in two ways: Either the gross tumor volume directly abutted the central airway or the PTV overlapped the trachea or main bronchi [21, 22].

In contrast, tumors which were immediately adjacent to the mediastinum and aorta had a higher acceptance for SBRT of 81-85% (case 3, 7 & 9). A reduced level of acceptance was also observed for cases where the tumor diameter exceeded 4 cm (case 2, 4 and 8).

SBRT was rejected by almost all radiation oncologists in case 4: The tumor exceeded 6 cm in diameter in almost every dimension and extensively infiltrated the right major bronchus up to the carina.

**Fig. 1:**
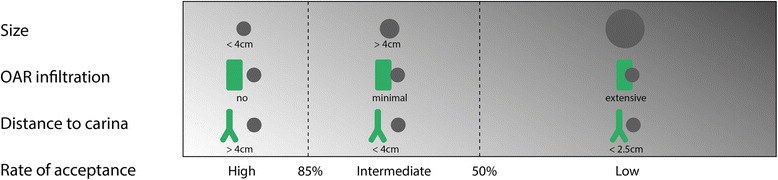
Criteria influencing the decision for or against SBRT

Taken together, the most prominent contraindications for SBRT were proximity to the carina, possible infiltration of the central airways (tumor immediately adjacent to the main bronchus) and tumor size beyond 4 cm. Each of the three criteria influenced the decision for or against SBRT independently, which is demonstrated in Fig. 1.

About half of the centers used schedules of eight fractions as their standard SBRT regimen for central lung tumors (Table 2), which was also the most frequently used regimen in the presented cases.

Sixty Gray in eight fractions is a common scheme for centrally located lung tumors propagated in particular by the group from VU University Medical Center Amsterdam/Netherlands [20, 23, 24]. The latest update of their series retrospectively compared 80 cases of central SBRT treated with eight fractions of 7.5 Gy (PTV overlapping a 2 cm expansion of the PBT according to RTOG 0813) to 252 cases of peripheral tumors treated with more escalated regimens [23]. There was no significant difference in overall survival between both groups and grade three pulmonary toxicity was observed in 6.4% of cases. In five cases the PTV overlapped the PBT, but only one patient developed a bronchial stricture and consecutively atelectasis. Although no grade four toxicity was described, six cases (7.5%) were considered to have possible (*n* = 3) or likely (*n* = 3) treatment-related death. Low toxicity rates of this SBRT regimen were also seen by Taremi et al. and Guckenberger et al., where two of twenty-two patients with central tumors developed grade two/three toxicity after irradiation with 48 Gy in eight fractions prescribed to the 65% isodose [25, 26]. As these data were not collected prospectively and patient cohorts show high pulmonary and cardiologic comorbidity, some toxicities may have escaped documentation. Furthermore, there may have been a low number of patients at risk for specific toxicities based on the dose distribution.

Five fraction regimens as second most common SBRT schedules were recommended in the intermediate acceptance group. More aggressive three fraction regimens were recommended only for tumors below 4 cm in size and distal of the PBT (high acceptance group). No center chose single-fraction radiotherapy.

It is important to mention that presented CT images were static. Additional information on tumor motion and corresponding organs at risk certainly would have influenced the decision whether and how SBRT would have been carried out. Obviously, this survey does not include the results of patient-specific interdisciplinary case discussion and counseling. Furthermore, not all participants completed the whole questionnaire. Due to the small number of participants the statistical analysis is descriptive rather than inferential.

## Conclusion

This survey showed a high level of acceptance of SBRT for selected cases with small centrally located tumors and minimal distance to the main bronchus of at least 2 cm. Apart from tumor size, the exact location within the central zone and the relation to critical organs at risk were important decision criteria for or against SBRT. Despite contact or even infiltration of the mediastinum, SBRT was accepted in small tumors not abutting the main bronchi. Acceptance of SBRT clearly decreased if the maximal tumor diameter exceeded 4 cm and the minimal distance to the main bronchus was below 2 cm. Secondly, we also observed a risk adaptation of SBRT fractionation within centrally located tumors, which was practiced in all participating institutions: higher risk tumors in terms of size and location were treated with a larger number of SBRT fractions and lower total SBRT doses. However, the variety of opinions on treatment of larger and more central tumors reflects the current controversy on this topic, which will hopefully be solved by running prospective clinical trials.

## Abbreviations

Cor: Coronal
CT: Computed tomography
DEGRO: Deutsche Gesellschaft für Radioonkologie e.V. (German society for radiation oncology)
FDG-PET: Fluordeoxyglucose positron emission tomography
Fr.: Fractions
IASLC: International Association for the Study of Lung Cancer
Long: Longitudinal
NSCLC: Non-small cell lung cancer
PBT: Proximal bronchial tree
PTV: Planning target volume
RT: Radiation therapy
RTOG: Radiation Therapy Oncology Group
Sag: Sagittal
SBRT: Stereotactic body radiation therapy
